# The first prospective application of AIGS real-time fluorescence PCR in precise diagnosis and treatment of meningioma: Case report

**DOI:** 10.3389/fnins.2023.1158601

**Published:** 2023-04-13

**Authors:** Zhe Han, Huizhong Chi, Xueen Li, Deze Jia, Haiyan Li, Shilei Ni, Kailiang Zhang, Zichao Feng, Qingtong Wang, Hao Xue, Gang Li

**Affiliations:** ^1^Department of Neurosurgery, Qilu Hospital, Cheeloo College of Medicine, Shandong University, Jinan, Shandong, China; ^2^Institute of Brain and Brain-Inspired Science, Shandong University, Jinan, Shandong, China; ^3^Shandong Key Laboratory of Brain Function Remodeling, Jinan, Shandong, China

**Keywords:** TERTp, PCR, intraoperative, surgery, molecular diagnosis, meningioma

## Abstract

**Background:**

The emergence of the new WHO classification standard in 2021 incorporated molecular characteristics into the diagnosis system for meningiomas, making the diagnosis and treatment of meningiomas enter the molecular era.

**Recent findings:**

At present, there are still some problems in the clinical molecular detection of meningioma, such as low attention, excessive detection, and a long cycle. In order to solve these clinical problems, we realized the intraoperative molecular diagnosis of meningioma by combining real-time fluorescence PCR and AIGS, which is also the first known product applied to the intraoperative molecular diagnosis of meningioma.

**Implications for practice:**

We applied AIGS to detect and track a patient with TERTp mutant meningioma, summarized the process of intraoperative molecular diagnosis, and expounded the significance of intraoperative molecular diagnosis under the new classification standard, hoping to optimize the clinical decision-making of meningioma through the diagnosis and treatment plan of this case.

## Introduction

Meningiomas have the highest incidence rate (39%) among all primary intracranial and central nervous system tumors. According to a report released in 2021 by the Central Brain Tumor Registry of the United States, the annual age-adjusted incidence rate of meningiomas in the United States (US) was 9.49 per 100,000 population in 2014–2018. The incidence increases with age, with a strong increase after the age of 65 years (Ostrom et al., 2021). The WHO classification of central nervous system tumors, published in 2021, confirmed that any meningioma with a telomerase reverse transcriptase gene promoter (TERTp) mutation and/or CDKN2A or B homozygous deletion, regardless of its histological characteristics, was classified as WHO grade 3 (Louis et al., 2021). This means that some meningiomas with histological diagnoses of grades 1 and 2 are degraded in treatment due to a lack of molecular diagnostic information, which affects the progression-free survival and overall survival of patients. At present, there are only a few molecular markers that are proven to be related to the prognosis of meningiomas and have diagnostic significance. Therefore, in the actual clinical diagnosis and treatment process, many neurosurgeons and pathologists have a low understanding of molecular diagnosis and still use the diagnostic criteria of histology, resulting in many patients being unable to achieve an accurate diagnosis and individualized treatment. However, due to the differences in the degree of development of various regions, the popularity of molecular testing is high in some regions, but we found that there are many detection sites with less significance under the commercialized detection package, which not only wastes medical resources but also increases the economic burden on patients. At present, the common detection method is postoperative sequencing, which takes a long time, and the average result feedback time is 10 days. Therefore, based on the above clinical problems, we designed a set of products for intraoperative molecular diagnosis. Through the automatic integrated gene detection system (AIGS) and real-time fluorescence PCR technology, the product can feed back the TERTp mutation information of patients within 1 h, realizing individualized and accurate intraoperative diagnosis and treatment under the new classification standard. In this paper, we describe the diagnosis and treatment of a patient with TERTp mutant meningioma and hope to optimize the clinical decision-making process for meningioma.

## Case summary

### Diagnosis and treatment process

**Figure 1:**
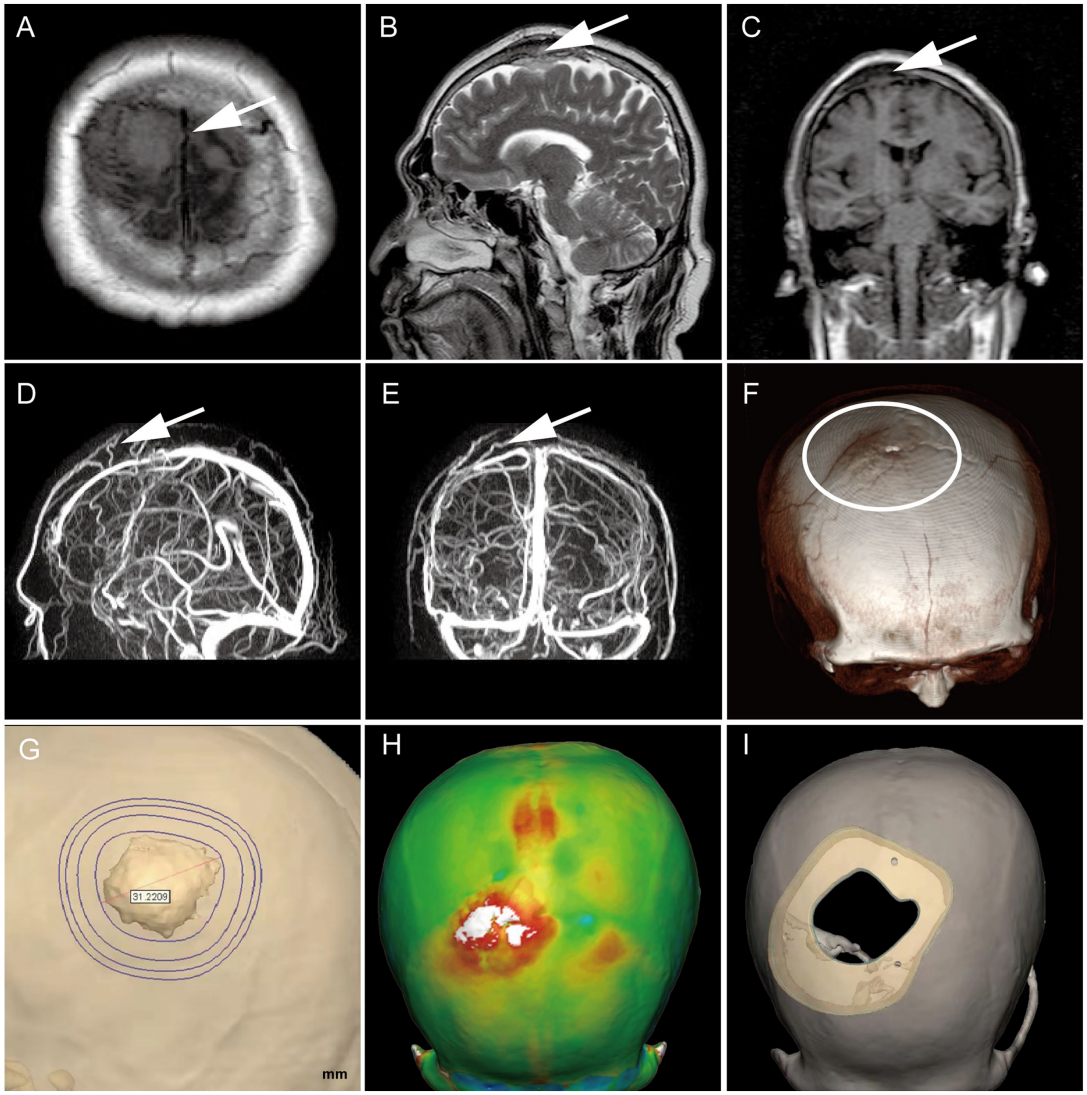
Preoperative MRI confirmed the tumor’s location (white arrow), with T1 low signal and T2 equal high signal, invading the skull and compressing brain tissue locally **(A–C)**.MRV suggests that it is closely related to the branches of the sagittal sinus **(D,E)**; Preoperative CT three-dimensional reconstruction showed a slight skull bulge at the lesion site **(F)**; Determine the skull repair scheme by three-dimensional modeling **(G–I)**.

**Figure 2:**
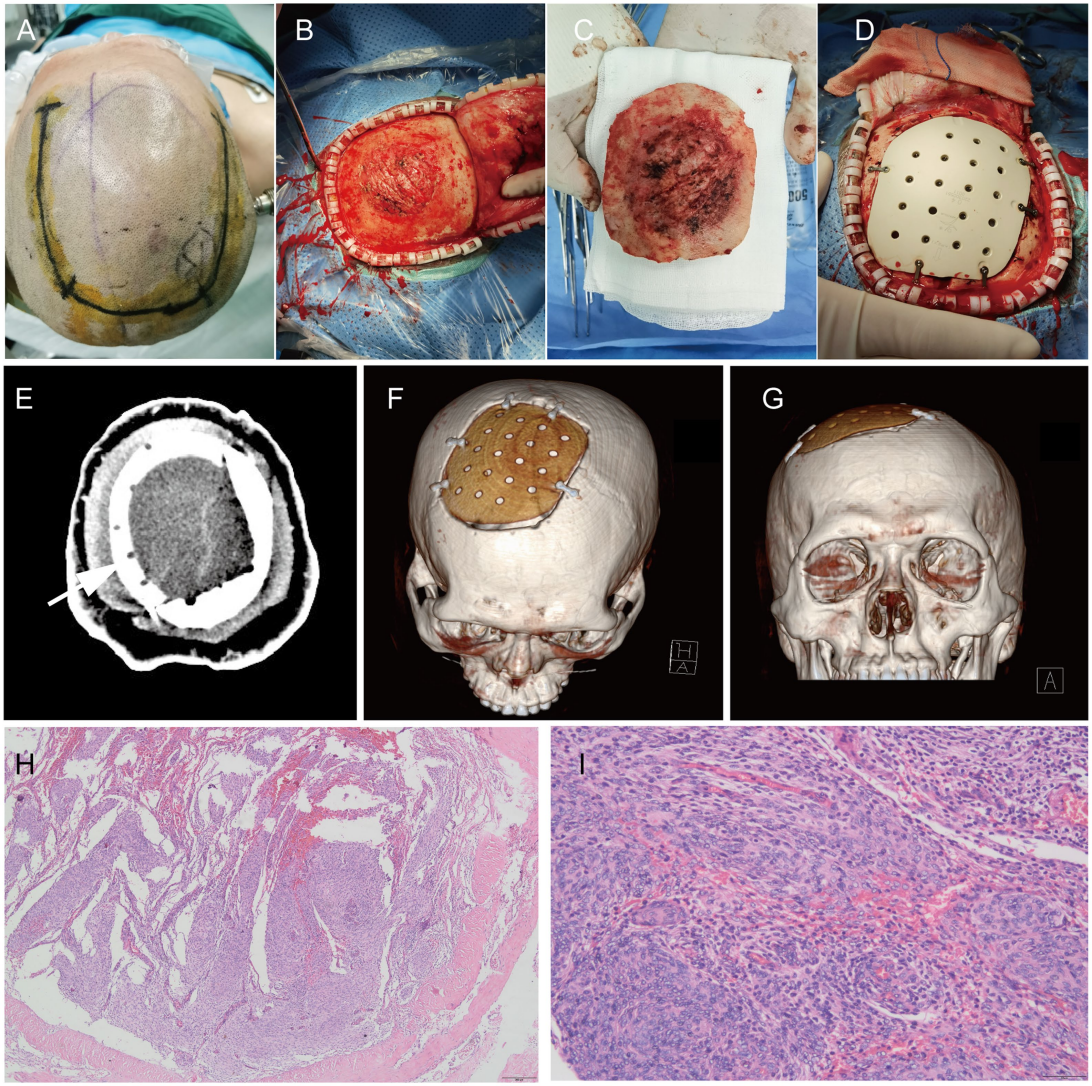
During the operation, the scalp incision range was planned **(A)**. After cutting the periosteum, the outer plate of the skull was rough and convex **(B)**. After removing the bone flap, the inner plate of the skull was severely eroded **(C)**. PEEK material was used for repair **(D)**; CT plain scan **(E)** and three-dimensional reconstruction **(F,G)** were performed after operation; H&E staining pictures of pathological tissues of meningiomas after operation: 40x **(H)** and 200X **(I)**.

On 23 March 2022, a 54-year-old male patient was treated for half a year due to paroxysmal headache. MRI and MRV examinations were performed on the same day, which showed that there were solid space-occupying lesions near the right frontal midline that were closely related to the superior sagittal sinus, mild compression of brain tissue, and local skull invasion (Figures 1A–E). According to previous experience, considering that meningioma invaded the skull, a CT plain scan and three-dimensional reconstruction were carried out to determine the invasion location and plan the skull repair area (Figures 1F–I). On 29 March 2022, the patient underwent resection of the right frontal tumor. The scalp was cut layer by layer. The skull at the lesion site was slightly raised. After milling the bone flap, the inner plate of the skull was involved and proliferated. After cutting the dura mater along the edge of the tumor, the tumor was gray, the boundary was clear, and there was a small amount of adhesion with the brain tissue. The dura mater of the tumor and surrounding lesions was completely removed, and the affected skull was removed at the same time, and the skull was remodeled with PEEK material (Figures 2A–D). Three days after the operation, we performed a CT scan and three-dimensional reconstruction to observe the intracranial condition and skull repair (Figures 2E–G). The histopathological examination 4 days after the operation revealed that meningioma, WHO grade 2, was absent and no lesion was found at the cutting edge (Figures 2H,I). That is, the operation reached the Simpson I resection. The patient recovered well after the operation and was discharged on 15 April 2022.

### Intraoperative molecular diagnosis

**Figure 3:**
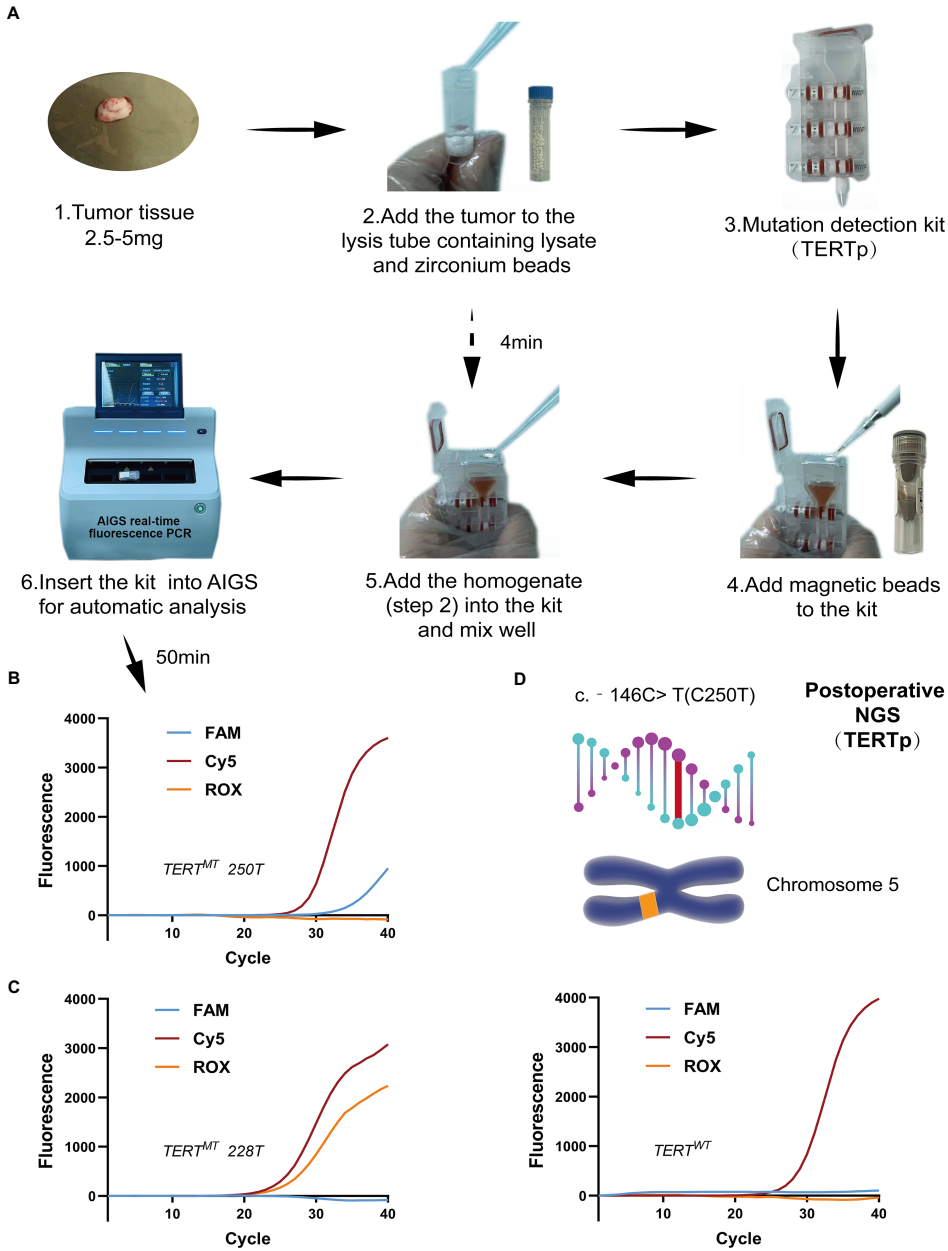
AIGS real-time fluorescence PCR was used for intraoperative molecular diagnosis **(A)**. Real-time fluorescence curve **(B)** represents TERTp C250T mutation (FAM and Cy5 showed S-type amplification). NGS was performed at the same time after operation. The results showed that the patient had TERTp C250T mutation **(C)**; Panel **(D)** represents the meaning of the other two curves detected by AIGS. (Left): TERTp C228T mutation (ROX and Cy5 showed S-type amplification), (Right): TERTp wild type (Cy5 showed S-type amplification). FAM is the C250T probe, Cy5 is the GAPDH internal reference probe, and ROX is the C228T probe.

Since it is clearly pointed out in the 2021 WHO classification standard for meningiomas, regardless of its histological diagnosis of grade 1, grade 2, or grade 3, as long as there is a TERTp mutation and/or CDKN2A/BB homozygous deletion, it is defined as WHO grade 3 (Louis et al., 2021). Therefore, in order to clarify the mutation status, we used AIGS real-time fluorescence PCR to detect the TERTp mutation during operation (Figure 3A). Take a 2.5–5 mg isolated meningioma sample, add it to the lysis tube, shake it, centrifuge it for 4 min, take the homogenate, add it to the self-designed integrated detection kit, and insert it into the AIGS card slot. The results are given after 50 min. The FAM curve is the C250T mutation (Figure 3B), ROX is the C228T mutation (Figure 3C), and the patient is the C250T mutation. After feeding back the results to the surgeon, he consciously expanded the resection scope of the dura mater based on the original resection and provided a theoretical basis for skull remodeling rather than electrocautery. Patients underwent NGS after surgery to demonstrate the accuracy of detection, and the results indicated the presence of the TERT C250T mutation (Figure 3D). According to the molecular mutation of this patient, the final integrated diagnosis result is: meningioma, TERTp mutation, WHO grade 3. Therefore, at the time of discharge, we provided patients with a more active follow-up adjuvant treatment scheme. Although Simpson I resection was achieved, fractionated radiotherapy (RT) was still necessary, and reexamination should be more frequent. On 9 May 2022, the patient came to the clinic for reexamination, and the imaging examination showed a normal postoperative state. The patient was generally in good condition and was preparing for radiotherapy. The case study was approved by the ethics committee of Qilu Hospital of Shandong University, and the patient provided written, informed consent.

## Discussions

Meningiomas are mostly benign tumors and grow slowly. However, some meningiomas relapse, often accompanied by an improvement in grade; most of these meningiomas have specific gene mutations (Saraf et al., 2011). Many studies have shown that the TERTp mutation is associated with poor prognosis and the invasiveness of tumors in meningiomas (Sahm et al., 2016; Mirian et al., 2020). The meta-analysis of Lu et al. found that the TERTp mutation occurred in 8% of meningiomas, and the incidence rates in WHO grade 1, 2, and 3 meningiomas were 1%, 6%, and 14%, respectively. Mortality increased by 3.79 times in the mutant population, and overall survival was cut by 5 years (Lu et al., 2019). Therefore, the revision of molecular markers in the 2021 WHO classification standard replaces the histological classification and grading standards of many brain tumors. For meningiomas, TERTp mutation and/or CDKN2A/B homozygous deletion are regarded as the independent standards for grading grade 3 meningiomas (Louis et al., 2021). There were significant differences in survival and prognosis between WHO grade 3 meningiomas and grade 1 and grade 2 meningiomas. Among 7,811 WHO grade 2 patients and 1936 WHO grade 3 meningiomas obtained from the national cancer database from 2004 to 2014, the 5-year overall survival rate (OS) of grade 2 patients was 75.9% and that of grade 3 meningiomas was 55.4% (*P* < 0.0001; Rydzewski et al., 2018).

As a result, understanding how to accurately grade meningiomas using molecular detection is critical, as it not only affects patients’ follow-up adjuvant treatment plans but also predicts their survival and prognosis. At present, the mainstream detection method in the market is NGS, but in the actual clinical work, it is found that the average time of this detection is 10 days, and the results cannot play a role in the intraoperative surgical decision-making. In the guidelines for the diagnosis and treatment of meningiomas issued by the European Association of Neuro-Oncology (EANO; Goldbrunner et al., 2021), it is suggested that for WHO grade 1 meningiomas with high surgical risk, some residual tumors are allowed after weighing, and the residual tumors may not need immediate radiotherapy. Because the recurrence time of WHO grade 3 meningiomas is short and the survival prognosis is poor, radical surgery should be performed as soon as possible. Simpson I resection should be performed as far as possible after it is determined to be WHO grade 3 meningioma during operation to prolong the recurrence time and avoid reoperation. At the same time, in clinical work, we also found that many gene points in commercial NGS detection have little relationship with the typing and prognosis of meningiomas, which not only wastes detection resources but also increases the economic burden of patients. Based on the current research, among many mutation sites, the KLF4/TRAF7 mutation can be used to diagnose secretory meningiomas (Clark et al., 2013; Williams et al., 2019; Youngblood et al., 2021). AKT1 and SMO mutations are related to a better prognosis for meningiomas (Yuzawa et al., 2016; von Spreckelsen et al., 2020). But only TERTp and/or CDKN2A or B can be used for meningioma grading. Therefore, based on the many clinical problems listed above, we have successfully transformed laboratory technology into clinical application by combining AIGS and real-time fluorescence PCR detection and designing the TERT mutation detection kit. In the preliminary study (Chinese Clinical Trial Registry: ChiCTR2100048172), we have evaluated the sensitivity, specificity, and accuracy of the detection technology, and the detection results are completely consistent with the results of the NGS. It can complete the automatic interpretation of the results within 1 h, and the accuracy rate is up to 100%,which can be applied in the operation (Xue et al., 2022). In our case, it is precisely because of the TERTp mutation information provided by AIGS that we can diagnose WHO grade 3 meningioma during operation. Therefore, we consciously performed an extended resection of the dura mater based on the original resection and decisively removed the invading skull and remolded it. Although no obvious histological features of grade 3 meningioma were found in postoperative histopathology, which was more inclined to grade 2, this did not hinder the diagnosis of WHO grade 3 meningioma.

EANO clearly stated in the guidelines for the diagnosis and management of meningiomas that molecular diagnosis of meningiomas is strongly recommended, which is not only related to accurate classification but also plays an important role in the treatment decision of meningiomas (Goldbrunner et al., 2021). At present, intraoperative molecular diagnosis has been applied to breast cancer, gastric cancer, lung cancer, etc. (Tamaki et al., 2009; Shimizu et al., 2012; Namba et al., 2022) providing a theoretical basis for optimizing the operation plan, determining the resection scope, and defining the subtype. However, the concept of intraoperative molecular diagnosis of central nervous system tumors has not been deepened. In the currently known research, we are the first team to develop intraoperative molecular diagnosis of meningiomas, and we have realized the transformation from basic to clinical research through early research. Therefore, due to the realization of intraoperative rapid molecular diagnosis technology, we believe that during the operation of meningioma resection, some tumor tissues should be taken for molecular diagnosis. When the detected tumor contains the TERTp mutation, the operation strategy should be adjusted and a more active resection scheme should be adopted. The bone tissue and dura that could have been retained should be removed together, and the resection scope should be expanded as much as possible. When it adheres to brain tissue, part of the brain tissue should be removed. AIGS real-time fluorescence PCR detection can be applied not only to meningiomas but also to gliomas, because our detection kit also includes the detection of the most important IDH mutation in gliomas. We believe that soon, the popularization of intraoperative molecular diagnosis technology will optimize the intraoperative treatment strategies for meningiomas, gliomas, and other brain tumors.

## Limitations

The detection time is a critical factor in the surgical process. Even though we can complete the detection in 1 h, strict surgical procedures may have an effect on the process. As a result, we have upgraded the next generation of AIGS products to further reduce detection time (within 35 min), and the sample size for synchronous detection has been expanded (16 detection channels) to meet different surgical needs while minimizing the impact on the surgical process. As a case report, this study cannot provide a clear conclusion on the level of evidence-based medicine, which would necessitate increasing the sample size and conducting long-term follow-up to assess prognosis. In addition, our team is conducting research in this area.

## Conclusion

In this case study, AIGS real-time fluorescence PCR was used for intraoperative molecular diagnosis of TERTp mutant meningioma. As the first product applied to intraoperative molecular diagnosis of meningioma, it has important pioneering significance. Through this technology, TERTp mutation can be accurately judged during operation, allowing us to adjust the operation plan in real time, optimize the treatment strategy, and provide important evidence support for intraoperative targeted treatment in the future. Finally, we strive to improve the survival and prognosis of patients.

## Patient perspective

Now is the 10th month after the operation. The patient came to the clinic for re-examination. From the imaging performance, the patient recovered well without any sign of recurrence. The patient was very satisfied with the surgical results and expressed his affirmation on the application of intraoperative molecular diagnosis and judgment technology to the determination of meningioma nature. Finally, the patient was also very happy to help more people with his diagnosis and treatment through case sharing.

## Data availability statement

The original contributions presented in the study are included in the article/supplementary material, further inquiries can be directed to the corresponding authors.

## Ethics statement

Ethical approval was not provided for this study on human participants because this study does not interfere with the normal diagnosis and treatment of patients. As a case report, it is important to record the whole diagnosis and treatment process of patients. The patients/participants provided their written informed consent to participate in this study. Written informed consent was obtained from the individual(s) for the publication of any potentially identifiable images or data included in this article.

## Author contributions

HX and GL designed and concepted of the study. ZH drafted the manuscript. HX revised the manuscript. HL, XL, DJ, KZ, and SN analyzed and interpreted of the data. ZH, HC, QW, and ZF played the role in the acquisition of data. All authors contributed to the article and approved the submitted version.

## Funding

This work was supported by the research on the future application prospect of classical gene marker Kit (contract no. 6010122006), Key Clinical Research Project of Clinical Research Center of Shandong University (2020SDUCRCA011), and Taishan Pandeng Scholar Program of Shandong Province (no. tspd20210322).

## Conflict of interest

The authors declare that the research was conducted in the absence of any commercial or financial relationships that could be construed as a potential conflict of interest.

## Publisher’s note

All claims expressed in this article are solely those of the authors and do not necessarily represent those of their affiliated organizations, or those of the publisher, the editors and the reviewers. Any product that may be evaluated in this article, or claim that may be made by its manufacturer, is not guaranteed or endorsed by the publisher.
